# Breast cancer screening in women taking hormone replacement therapy needs updating

**DOI:** 10.52054/FVVO.16.1.001

**Published:** 2024-03-28

**Authors:** P.R. Koninckx, A Ussia, G Page

**Affiliations:** Prof. emeritus Obstetrics and Gynecology KULeuven, Leuven, Belgium, the University of Oxford, Oxford, UK, University Cattolica, Rome, Italy and Moscow State University, Moscow, Russia; Gruppo Ialo Belga, Villa del Rosario, Rome Italy; OBGYN, MSc EBHC, Coordinator Clinical Guidance project VVOG, Belgium

## Abstract

Breast cancer screening by mammography is widely used. The diagnostic accuracy is limited, with a positive predictive value of 16%. Therefore, a stepwise investigation, with repeat mammography and confirmation by pathology, is usually proposed. Although this stepwise investigation intends to avoid overtreatment, the many false positives result in unnecessary fear and diagnostic surgery in many women. The false negatives are not known since these women have not been investigated. Given the estimated low risk of missing breast cancer and the slow growth, repeating a screening mammography every two years is sufficient.

## Introduction

**Figure 1:**
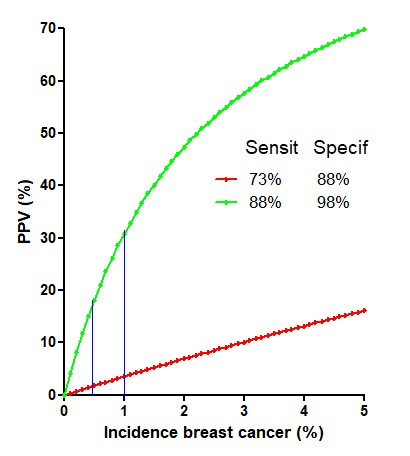
Positive predictive values (PPV= sensitivty*prevalence /(sensitivity*prevalence + (1-specificity)(1-prevalence)) of a mammography if sensitivities and specificities are 73% and 88% or 80% and 98%, respectively. This illustrates the crucial importance of the specificity and of the prevalence of the disease (0.5 and 1% indicated).

Mammography is widely used for breast cancer screening. The risk of overdiagnosis, overtreatment, and the many factors involved (Ryser et al., 2022), and the benefits measured by the estimated lifetime gained (Bretthauer et al., 2023) have been widely discussed. Less emphasised is that the reported sensitivities and specificities, varying between 73% and 88% (Schünemann et al., 2020) to 80% and 98% (Mushlin et al., 1998), result, at best, in positive predictive values of less than 5% to 18% for prevalences of 0.5% in the screened population (Figure 1). This is also illustrated by the Belgian breast cancer screening program with biannual mammography in women between 50 and 69 years old, following The European quality assurance program (Schünemann et al., 2020). Data from 2017 to 2020 illustrate that 5% of women screened had a second mammography, and 2% had more exams, such as MRI or biopsies, to detect between 0.5% and 0.6% breast cancers. Thus, out of 200 women screened, 10 are selected for a second mammography and 4 for more invasive exams to find 1 cancer.

This stepwise procedure compensates for the low predictive values of mammography with sensitivities and specificities of 67 % and 98% when prevalences are low (Koninckx et al., 2023). Sensitivities and specificities are test characteristics, while clinically, it is important to know the risk of having breast cancer if the test is positive and the risk of missing cancer when the test is negative. These are the predictive values, which decrease sharply when prevalences are less than 10%, the PPV being the sensitivity*prevalence /(sensitivity*prevalence + (1-specificity)(1-prevalence) ) (Lesaffre and Lawson, 2012). Therefore, excellent sensitivities and specificities around 80% and 98% result in poor predictive values when prevalences are 0.5%.

Although the interpretation of mammographies and the definition of abnormal findings or suspicious lesions seem to be established (Gøtzsche and Jørgensen, 2013), there is little consensus about screening ages and intervals between screening (Schünemann et al., 2020). Without discussing ductal carcinoma in situ, regarding the clinical benefits of screening or contrast mammography, we must realise the difficulties of performing randomised controlled trials for breast cancer screening and of translating the findings into guidelines. False and true negatives are poorly known since these women are not investigated. Evidence-based medicine and guidelines rely heavily on double-blind randomised controlled trials. However, RCTs are poorly suited to investigate multivariate events because of randomisation problems. A 2 or 3 Y/N factorial design already requires 4 or 8 groups. An RCT is not suited for rare events, and breast cancer trials require huge numbers taking time to perform with the risk of being outdated before being finished. This explains the lack of data on newer techniques in ultrasound, digital imaging, deep learning (Arun Kumar and Sasikala, 2023) and artificial intelligence (Gao et al., 2023). The translation of predictive values or Bayesian probabilities, into guidelines, results from estimating truth or clinical importance, decided by consensus or voting by a group of experts (Kubota et al., 2023), thus introducing subjectivity based on previous experiences. Therefore, guidelines may vary, although based on the same RCTs (Jhangiani et al., 2023). More fundamental is that accuracies and predictive values of abnormal or suspicious findings are crude estimates and should be stratified for factors such as breast densities, increasing the difficulty of interpretation and the risk of abnormal findings (Freer, 2015; Schünemann et al., 2020). Finally, although beyond this discussion, it should be realised that the accuracy of the diagnosis by pathology cannot be established since it is the gold standard, which cannot be compared with a better test.

In addition, it is unclear whether histopathological parameters reflect the expression profile or molecular genetic features (Simpson et al., 2005).

These poor predictive values of mammography and the stepwise clinical approach result in more lumpectomies and mastectomies (Gøtzsche and Jørgensen, 2013; Schünemann et al., 2020) with an estimated 30% overdiagnosis. Without discussing whether screening results in a reduction in breast cancer mortality, we should realise that a reduction of breast cancer mortality by 15% and an overdiagnosis and overtreatment of 30% results in preventing one mortality for every 2000 women screened for ten years, but at the cost of 10 women being treated unnecessarily, and 200 women having experienced psychological distress and anxiety. Although the exact figures can be discussed, the conclusion of overdiagnosis and little effect on mortality seems repetitively confirmed (Bretthauer et al., 2023).

Each management of a woman with endometriosis can be considered a unique experiment of diagnosis and treatment with an outcome, which is a complex and multivariate process. However, the clinician will instinctively continue successful management and update or change less successful ones. This progressive update of knowledge by new data to predict the future is fundamental in Bayesian statistics (Lesaffre and Lawson, 2012). Similarly, experience in managing endometriosis is updated by the result of each new management. However, in EBM and the pyramid of evidence, the experience of the individual clinician has been considered a personal opinion of low value (Djulbegovic and Guyatt, 2017) because of the many potential biases. However, a similar collective experience of many clinicians, decreasing personal bias, has more value than a personal opinion.

Over the last decades, traditional statistical analysis with significances and p-values (Fisher 1925; Neyman and Pearson, 1928) has been complemented with Bayesian statistics. The null hypothesis of traditional or frequentist statistics is that there is no difference between groups. The analysis evaluates the probability that an eventual difference can be explained by chance, and a probability of less than 5% is considered significant. Traditional statistical analysis thus can only refute but cannot confirm a hypothesis (Wasserstein and Lazar, 2016). This mistake, frequently made in biomedical research, is called the P-value fallacy (Goodman, 2001). Bayesian statistics, on the contrary, explores the probability that a hypothesis or an observed difference is true, using new data to update all previous data (the prior) (Lesaffre and Lawson, 2012). Bayesian statistics emphasises the uncertainty of whether a hypothesis is correct.

Frequentist and Bayesian analyses are related, and a p-value of 0.05 increases the probability of truth from 50% to some 70% (Nuzzo, 2014).

Therefore, to evaluate experience, a group of clinicians with experience in treating endometriosis were asked how they managed some aspects of the disease. Since management is based upon knowledge and a progressive updated experience by learning from the past, we planned to estimate the similarity of experiences in a group of clinicians. Without personal observer bias, this collective experience, based on all previous treatments, literature and discussions, will probably have more value than a personal opinion. The estimation of collective experiences was conceived as a proof of concept to establish a Bayesian prior, permitting subsequent statistical updates and the calculation of the probability that the statements are true.

## Breast cancer screening, clinical medicine and Bayesian statistics

Clinical medicine is multivariate, and in the individual woman (Koninckx et al., 2023), the probability of having breast cancer varies not only with the image of mammography (or MRI) but also with the clinical exam, age, heredity, obesity, breast density, race and many other factors. The estimation of the probability in the individual woman that an abnormal or suspicious lesion is a cancer should include all these factors besides the mammography. This requires multivariate Bayesian statistics, but these results are unfortunately not available.For example, since multivariate RCTs are difficult to perform, the added value of mammography following a negative clinical exam, with a reported sensitivity of 54% and specificity of 94% (Jatoi, 2003), is still unknown. Therefore, the translation of an abnormal or suspicious lesion into the management of the individual woman remains clinical judgment. This explains why recommendations for the diagnostic workup of abnormal or suspicious breast lesions vary.

## Breast cancer screening and hormone replacement therapy

Hormone replacement therapy (HRT) increases breast density and thus the risk of finding abnormal and suspicious lesions (Rutter et al., 2001; Freer, 2015; Azam et al., 2018). Therefore, it was suggested that HRT be stopped for a few months before screening mammography is performed (Beckmann et al., 2013). Unfortunately stopping HRT for several months is clinically poorly accepted. Since breast cancer screening by mammography is recommended to be performed only every two years, the risk of delaying the workup and eventual treatment of abnormal or suspicious lesions for 3 to 6 months must be very low. Therefore, managing women on HRT and abnormal or suspicious lesions on screening mammography must be clinically individualised. Considering all risk factors, the increase of abnormal or suspicious images in women taking HRT and the low risk of delaying therapy for a few months, it may be considered to stop HRT for 3 to 6 months before repeating mammography. The women whose repeat mammography returns to normal will have avoided unnecessary cancer workups and surgery, and the continuation of HRT can be discussed.

## Conclusion

The limitations of performing multivariate RCTs and the frequentist statistical analysis hamper the interpretation of breast cancer screening by mammography. It is still not fully appreciated that the predictive value of any test is strongly affected by the prevalence of the disease (Lesaffre and Lawson, 2012). For breast cancer with a prevalence of 0.5%, the positive predictive value is only 16% for sensitivities of 67% and specificities of 98%. Unfortunately, negative predictive values cannot be estimated since the women in whom breast cancer is missed (false negatives) are unknown since they have not been further explored. Also, although estimated as high as 40% (Ryser et al., 2022), the true incidence of false positives is not clear since expectant management of suspicious lesions is considered unethical. Clinically important is that frequentist statistical analysis calculates the positive predictive values of breast cancer screening but not the added value besides age, heredity and breast density. This would require a Bayesian statistical approach, which has not been performed yet to the best of our knowledge.

The diagnostic workup of abnormal and suspicious lesions follows a clinical logic of repeat exams and, eventually, biopsies, with the dogma (without data) that delaying the workup and treatment can only be harmful. Overtreatment and unnecessary fear have been discussed, but the hypothesis that some lesions could disappear spontaneously, especially in women stopping HRT, has not been tested.

It is beyond this manuscript to discuss the limitations of frequentist statistics or the need to individualise therapy and to consider all predictive factors or medical and corporate pressures, as we recently did for endometriosis (Koninckx et al., 2022). However, individualising follow-up and postponing exams for three months in women with abnormal or suspicious lesions and dense breasts while HRT is stopped has the potential to decrease overtreatment. Although it would be an easy trial to demonstrate that some abnormal images become normal after stopping HRT for three months, it is close to impossible to demonstrate that a delay of 3 months does not increase the risk since proving the absence of an effect requires huge numbers. Therefore, an adequately performed RCT to demonstrate a decreased risk of unnecessary treatments without increasing risks is unlikely to be performed. In conclusion, individualising follow-up and letting the woman decide whether to stop HRT for three or more months before repeating mammography has the potential to decrease overtreatment.
